# Content Factor: A Measure of a Journal’s Contribution to Knowledge

**DOI:** 10.1371/journal.pone.0041554

**Published:** 2012-07-23

**Authors:** Joseph Bernstein, Chancellor F. Gray

**Affiliations:** 1 Department of Orthopedic Surgery, Veterans Hospital, Philadelphia, Pennsylvania, United States of America; 2 Department of Orthopedic Surgery, University of Pennsylvania School of Medicine, Philadelphia, Pennsylvania, United States of America; University of Pittsburgh Medical Center, United States of America

## Abstract

Impact Factor, the pre-eminent performance metric for medical journals, has been criticized for failing to capture the true impact of articles; for favoring methodology papers; for being unduly influenced by statistical outliers; and for examining a period of time too short to capture an article’s long-term importance. Also, in the era of search engines, where readers need not skim through journals to find information, Impact Factor’s emphasis on citation efficiency may be misplaced. A better metric would consider the total number of citations to all papers published by the journal (not just the recent ones), and would not be decremented by the total number of papers published. We propose a metric embodying these principles, “Content Factor”, and examine its performance among leading medical and orthopaedic surgery journals. To remedy Impact Factor’s emphasis on recent citations, Content Factor considers the total number of citations, regardless of the year in which the cited paper was published. To correct for Impact Factor’s emphasis on efficiency, no denominator is employed. Content Factor is thus the total number of citations in a given year to all of the papers previously published in the journal. We found that Content Factor and Impact Factor are poorly correlated. We further surveyed 75 experienced orthopaedic authors and measured their perceptions of the “importance” of various orthopaedic surgery journals. The correlation between the importance score and the Impact Factor was only 0.08; the correlation between the importance score and Content Factor was 0.56. Accordingly, Content Factor better reflects a journal’s “importance”. In sum, while Content Factor cannot be defended as the lone metric of merit, to the extent that performance data informs journal evaluations, Content Factor– an easily obtained and intuitively appealing metric of the journal’s knowledge contribution, not subject to gaming– can be a useful adjunct.

## Introduction

Impact Factor, conceived by Garfield [1] and promulgated by Thomson Reuters’s *Journal Citation Reports*, is the pre-eminent performance metric for medical journals. The Impact Factor is defined as “a ratio between citations and recent citable items published. Thus, the Impact Factor of a journal is calculated by dividing the number of current year citations to the source items published in that journal during the previous two years” [2] by the total number of such source items. For example, the 2010 Impact Factor for the journal *CA: A Cancer Journal for Clinicians* was 94.33, the highest among all scientific journals. This number is calculated by noting that 19 source items were published in 2008 and 23 items in 2009 and in turn the journal’s 2008 and 2009 material was cited a total of 3,962 times in 2010 (3,962/42 = 94.33).

Impact Factor has its detractors. One criticism centers on that citations fail to capture “how well read and discussed the journal is outside the core scientific community or whether it influences health policy” [3]. (For example, the 2008 JAMA articles by Barack Obama [4] and John McCain [5] on health care reform have been cited only ten times as of this writing.) Further, because citations do not follow a normal distribution, Impact Factor can be “influenced by a small minority of [a journal’s] papers” [6]: for example, the impact factor for *CA-A CANCER JOURNAL FOR CLINICIANS* drops from 94.33 to 8.07 if the two papers cited most in 2010, namely “Cancer statistics 2008” and “Cancer statistics 2009”, are dropped from consideration.

Beyond the issue of whether counting citations is a valid method of measuring a journal’s impact, one may wonder if a two-, or even a five-, year-window is inadequate. Consider the paper by Warren [7] published in 1983 suggesting that peptic ulcer disease was caused by H. pylori. By 1985– the last year this paper could be counted toward *The Lancet’s* Impact Factor–it was cited 37 times. In the years that followed, the paper was cited more than 2,000 additional times, with profound impact on both the author (who won the 2005 Nobel Prize) and the practice of medicine. Second, in the modern era of search engines, the emphasis on citation efficiency implicit in the Impact Factor denominator may be misplaced. Because a search engine can scan millions of papers in an instant, readers are not hampered by the publication of lower yield material (as they were when they needed to wade through journals by hand). Why penalize a journal for publishing good science that is not cited frequently–as the Impact Factor does– if the addition of that paper adds no impediment to the uninterested readers?

**Table 1:** Content and Impact Factors for the twenty biomedical journals with highest Impact Factors, 2010, listed in order of highest Content Factor.

Journal	Content Factor	Impact Factor
1. NATURE	511.2	36.1
2. SCIENCE	469.8	31.4
3. NEW ENGL J MED	227.7	53.5
4. CELL	167.6	32.4
5. LANCET	155.7	33.6
6. JAMA-J AM MED ASSOC	117.5	30.0
7. CHEM REV	88.4	33.0
8. NAT GENET	76.3	36.4
9. NAT BIOTECHNOL	34.5	31.1
10. NAT MATER	32.0	29.9
11. REV MOD PHYS	29.9	51.7
12. NAT REV MOL CELL BIO	26.8	38.7
13. NAT REV CANCER	26.7	37.2
14. NAT REV IMMUNOL	21.1	35.2
15. ANNU REV BIOCHEM	18.6	29.7
16. NAT REV GENET	18.5	32.7
17. ANNU REV IMMUNOL	16.1	49.3
18. ACTA CRYSTALLOGR A	13.9	54.3
19. NAT NANOTECHNOL	11.4	30.3
20. CA-CANCER J CLIN	9.8	94.3

The Impact Factor is calculated by dividing the number of current year citations to source items published in the given journal during the previous two years by the total number of source items; the Content Factor is the total number of citations in a given year to all of the papers the journal had published up to and including the year in question, reported in “kilo-cites” (ie thousands of citations).

A better metric might consider the total number of citations in a given year to *all* papers published by the journal – not just the recent ones– to eliminate the bias against more slowly adopted science. This metric would also not be decremented by the total number of papers the journal published. We thus propose a new metric embodying these principles: the “Content Factor”. In this paper, we consider the Content Factors for the leading medical and orthopaedic surgery journals and we address the differences between Content Factor and Impact Factor especially as they relate to the perceived importance of the journal.

## Methods

Content Factor was defined using information provided by Science Citation Index. To remedy Impact Factor’s emphasis on only recent citations, the Content Factor considered the total number of citations to the journal in a given year, regardless of the year in which the cited paper was published. To correct for the Impact Factor’s emphasis on citation efficiency, no denominator was employed. The Content Factor, then, is simply the total number of citations in a given year to all of the papers the journal had published up to and including the year in question. The Content Factor is reported in kilo-cites (the total number of citations divided by 1000) to present units comparable in magnitude to those typically reported for Impact Factor.

For illustration, Content Factor was calculated for the general medical and orthopedic journals with the highest Impact Factor for the year 2010. The Impact Factor and Content Factor were correlated with the Pearson correlation factor. In addition, a survey was presented to the contributors to the Orthopedic Knowledge Update [8]. This text is published by the American Academy of Orthopaedic Surgeons, and the authors are selected for their expertise. These authors were asked to declare their perception of the “importance” of a sample of ten orthopedic journals (the 10 journals with the highest Impact Factor in 2009, the most recent data available when the survey was conducted). The authors were asked to assign an “importance score” to each of the ten journals, ranging from one to ten. A rank order list was not requested; two journals could be given the same score. The correlation between the mean importance score and the Content Factor and Impact Factor of the journals was assessed as well.

This study, examining journals and not people, was exempt from ethics committee review.

**Table 2:** Content and Impact Factors for the twenty orthopaedic surgery journals with highest Impact Factors, 2010, listed in order of highest Content Factor.

Journal	Content Factor	Impact Factor
1. SPINE	33.12	2.51
2. CLIN ORTHOP RELAT R	28.68	2.12
3. J BONE JOINT SURG AM	23.56	2.97
4. AM J SPORT MED	15.49	3.82
5. J BONE JOINT SURG BR	14.76	2.35
6. J ORTHOP RES	10.69	2.98
7. ARTHROSCOPY	8.59	3.32
8. OSTEOARTHR CARTILAGE	7.14	3.95
9. J ARTHROPLASTY	6.67	2.21
10. INJURY	6.30	2.27
11. PHYS THER	6.25	2.65
12. EUR SPINE J	5.18	1.99
13. CLIN BIOMECH	4.85	2.04
14. J SHOULDER ELB SURG	4.68	2.31
15. GAIT POSTURE	4.44	2.31
16. J ORTHOP SPORT PHYS	2.94	2.54
17. SPINE J	2.71	3.02
18. J AM ACAD ORTHOP SUR	2.26	2.55
19. CLIN J SPORT MED	2.21	2.11
20. CONNECT TISSUE RES	1.79	2.09

Content Factor is the total number of citations in a given year to all of the papers the journal had published up to and including the year in question, reported in “kilo-cites” (ie, thousands of citations). For example, the 2010 Content Factor for the journal *Clinical Orthopaedics and Related Research* was 28.68, meaning that in 2010 there were approximately 28,680 (28,676 to be precise) citations in the medical literature to papers that had (ever) been published in Clinical Orthopaedics and Related Research.

**Table 3:** Importance Scores Assigned by the OKU Authors to a Sample of 10 Orthopaedic Surgery Journals, along with their Content and Impact Factors.

Journal	Importance Score	Content Factor	Impact Factor
1. J BONE JOINT SURG AM	8.8	23.56	2.97
2. J BONE JOINT SURG BR	7.0	14.76	2.35
3. J ORTHOP RES	6.4	10.69	2.98
4. CLIN ORTHOP RELAT R	5.8	28.68	2.12
5. OSTEOARTHR CARTILAGE	5.7	7.14	3.95
6. SPINE	5.7	33.12	2.51
7. AM J SPORT MED	5.0	15.49	3.82
8. ARTHROSCOPY	4.1	8.59	3.32
9. PHYS THER	2.5	6.25	2.65
10. GAIT POSTURE	2.4	4.44	2.31

## Results

The Impact Factor and Content Factor of the twenty biomedical journals with highest Impact Factors for 2010 are shown in Table 1. The Pearson correlation between Impact Factor and Content Factor is –0.18.

The Impact Factor and Content Factor of the twenty orthopaedic surgery journals with highest Impact Factors for 2010 are shown in Table 2. The Pearson correlation between Impact Factor and Content Factor is 0.12.

Seventy–five of the 115 Orthopedic Knowledge Update authors (65%) completed the survey. One of the respondents was a resident in training; six had no academic affiliation. Of the 68 who remained, 25 were assistant professors, 24 were associate professor and 19 were full professors. Their average age was 44, and the mean number of published papers by this group was 63.9. The importance scores assigned by the authors to the sample of 10 orthopaedic surgery journals are shown in Table 3. The Pearson correlation between this score and the Impact Factor was 0.08; the Pearson correlation between the importance score and Content Factor was 0.56.

## Discussion

If the mission of biomedical journals is to add knowledge, and if citation is a reasonable means of measuring knowledge added, Content Factor can be a helpful metric for assessing success in that mission.

Content Factor is closely related to Impact Factor, but corrects for two features of the Impact Factor that may distort it: namely, that Impact Factor only considers citations to recent publications and that Impact Factor is lowered in proportion to the total number of papers the journal publishes. As such, Content Factor is a metric of total knowledge added, not only immediate knowledge; and Content Factor considers the total knowledge contribution, not the efficiency with which that contribution is made.

The main strength of Content Factor is that it is an intuitively appealing metric of the journal’s knowledge contribution: as shown, it more highly correlates with the journal’s importance, as deemed by a panel of experts. Content Factor, accordingly, can function more readily as a shorthand notation of “importance”. (Note that the importance scores were provided by academic surgeons. Different results might have been seen if different respondents were polled.).

Content Factor also is less amenable to being gamed by editors. An editor who aims to optimize the journal’s Impact Factor might reject a paper that is in all other ways excellent, but not apt to be widely cited. (That category might include not only papers on esoteric topics but also negative–result studies.) Also, because Thomson Reuters is said to deem certain “less substantial” pieces as non citable items and exclude them from the Impact Factor denominator, editors may also take steps to ensure that a piece is not counted as a source item “by making such articles superficially less substantial, such as by forcing authors to cut down on the number of references or removing abstracts” [3]–to the detriment of reader and writer alike.

An emphasis on Content Factor may lessen such editorial biases. Because every additional citation augments the Content Factor, there is no incentive to reject potentially unpopular papers; and because there is no count of “citable items,” there is no need to massage manuscripts into one category or another.

There are flaws one could find with Content Factor, to be sure, but the most obvious is that it dismisses elements of the Impact Factor that some might find desirable. For one thing, a journal gets Content Factor credit for all citations its papers earn, however old these papers may be. Such a scoring system may misrepresent the journal’s recent performance. (While Impact Factor is also hampered by statistical outliers, at least in the case of Impact Factor the outliers drop out of the calculation after two years.) Also, by failing to consider the total number of papers published, Content Factor may give a misleading sense that a reader will be rewarded for casual browsing.

Content Factor purports to measure only one thing: knowledge contribution, as reflected by citation. Those who use Content Factor as the sole determinant of a journal’s performance will have an incomplete picture. And of course it must be recalled that both Impact Factor and Content Factor are metrics of a journal’s performance *en toto*, over a period of years. Neither metric says anything about the quality of one particular article. If a reader wishes to know if a given article is any good, the best way to find out is to read it closely. Because close reading is hard work, it is understandable that the invocation of a single number replacing all of that hard work might be appealing. We argue that neither Impact nor Content Factor is an appropriate replacement for direct scrutiny of the work. Nonetheless, to the extent that performance data can help inform journal evaluation, Content Factor – an easily obtained metric, not subject to manipulation –can be a useful adjunct.
